# Antigen Specificity Enhances Disease Control by Tregs in Vitiligo

**DOI:** 10.3389/fimmu.2020.581433

**Published:** 2020-12-01

**Authors:** Zhussipbek Mukhatayev, Emilia R. Dellacecca, Cormac Cosgrove, Rohan Shivde, Dinesh Jaishankar, Katherine Pontarolo-Maag, Jonathan M. Eby, Steven W. Henning, Yekaterina O. Ostapchuk, Kettil Cedercreutz, Alpamys Issanov, Shikhar Mehrotra, Andreas Overbeck, Richard P. Junghans, Joseph R. Leventhal, I. Caroline Le Poole

**Affiliations:** ^1^ Department of Dermatology, Northwestern University, Chicago, IL, United States; ^2^ Robert H. Lurie Comprehensive Cancer Center, Northwestern University, Chicago, IL, United States; ^3^ Faculty of Biology and Biotechnology, Al-Farabi Kazakh National University, Almaty, Kazakhstan; ^4^ Laboratory of Molecular immunology and Immunobiotechnology, M.A. Aitkhozhin’s Institute of Molecular Biology and Biochemistry, Almaty, Kazakhstan; ^5^ Oncology Research Institute, Loyola University, Maywood, IL, United States; ^6^ Department of Medicine, School of Medicine, Nazarbayev University, Nur-Sultan, Kazakhstan; ^7^ Department of Surgery, Medical University of South Carolina, Charleston, SC, United States; ^8^ Department for Surgery of Pigment Disorders, Lumiderm, Madrid, Spain; ^9^ Department of Hematology/Oncology, Boston University, Boston MA, United States; ^10^ Comprehensive Transplant Center, Northwestern Memorial Hospital, Chicago, IL, United States

**Keywords:** vitiligo, regulatory T cells, chimeric antigen receptor T cells, ganglioside D3, antigen-specific Treg, autoimmune diseases

## Abstract

Vitiligo is an autoimmune skin disease characterized by melanocyte destruction. Regulatory T cells (Tregs) are greatly reduced in vitiligo skin, and replenishing peripheral skin Tregs can provide protection against depigmentation. Ganglioside D3 (GD3) is overexpressed by perilesional epidermal cells, including melanocytes, which prompted us to generate GD3-reactive chimeric antigen receptor (CAR) Tregs to treat vitiligo. Mice received either untransduced Tregs or GD3-specific Tregs to test the hypothesis that antigen specificity contributes to reduced autoimmune reactivity *in vitro* and *in vivo*. CAR Tregs displayed increased IL-10 secretion in response to antigen, provided superior control of cytotoxicity towards melanocytes, and supported a significant delay in depigmentation compared to untransduced Tregs and vehicle control recipients in a TCR transgenic mouse model of spontaneous vitiligo. The latter findings were associated with a greater abundance of Tregs and melanocytes in treated mice versus both control groups. Our data support the concept that antigen-specific Tregs can be prepared, used, and stored for long-term control of progressive depigmentation.

## Introduction

Vitiligo is an autoimmune disease wherein melanocytes are progressively destroyed, resulting in pale, white patches of skin (1, 2). The prevalence of vitiligo is 0.5–1%, and this disorder is associated with low self-esteem, psychological stress, and social stigma (3–5). Therapies currently available for vitiligo have limited efficacy, and a real need for new treatment strategies exists (6). Several factors contribute to the pathogenesis of vitiligo. Oxidative stress in melanocytes can trigger the release of inducible heat shock protein 70 (HSP70i) (7). HSP70i can directly activate innate immune cells and chaperone melanocyte antigens for subsequent introduction to antigen presenting cells (APCs) (8, 9). In turn, these innate immune cells and APCs recruit auto-reactive T cells to mediate specific destruction of melanocytes in vitiligo (10). The loss of tolerance to self-antigens also involves reduced cutaneous regulatory T cell (Treg) activity (11, 12). Tregs are subsets of T cells responsible for peripheral tolerance *via* suppression of immune cells, including self-reactive, cytotoxic T cells, to maintain immune homeostasis (13). Paucity of and defects in Tregs have been reported in several autoimmune disorders (14–16). A local deficiency in Tregs was found in vitiligo skin within lesional, non-lesional, and perilesional sections, thereby suggesting a reduced ability of vitiligo patients to maintain peripheral immune homeostasis (17, 18). Others have reported that Tregs may be circulating in reduced number or exhibit reduced activity (16, 19). Limited Treg activity in vitiligo skin allows autoreactive, cytotoxic T cells to expand and eliminate melanocytes from the skin, facilitating progressive depigmentation (12).

To date, adoptive transfer of polyclonal Tregs has been used in clinical trials for several conditions wherein autologous cells were amplified *in vitro* and re-administered to patients, with limited success (20). In a mouse model of vitiligo, this approach was beneficial, by short term-maintenance of a favorable Treg to effector T cell ratio in this setting (21). Application of polyclonal Tregs can however impart systemic immunosuppression, *via* inadvertent dampening of responses to infection or malignancies, posing an important clinical consideration (22). Furthermore, generating sufficient numbers of polyclonal Tregs for clinical use can be challenging. Generic immunosuppression can present an issue that might be overcome by including antigen specificity to adoptively transferred cells. In transplant biology, antigen-specific Tregs are showing promise to provide local immunosuppression at the site of disease progression by targeting human leukocyte antigen (HLA) discrepancies between donor and recipient (23). Antigen-specific Tregs might offer significant advantages for the treatment of vitiligo as well.

Antigen-specific Tregs can be generated by introducing T-cell receptors (TCRs), but applications of these TCR-Tregs are limited by major histocompatibility complex (MHC)-restriction, not allowing all patients to benefit (24). Our study instead makes use of antigen-specific Tregs transduced to express a chimeric antigen receptor (CAR), which overcomes MHC-dependency. In CAR-T cell therapy, optimizing and selecting the correct CAR affinity and intracellular signaling domains is particularly important for the resulting therapeutic activity and cellular persistence of the Tregs. Since CARs are constructed using antibody variable regions, they hold higher affinity to their cognate antigen compared to TCRs (25). Also important is the identification of a cell surface antigen that is relatively abundant in the affected tissue under study and can be targeted by CAR Tregs. The concept is then that the selected surface antigen can serve as a target for antigen-specific CAR Tregs to selectively and locally suppress auto-reactive T cells in vitiligo (26). These results can be compared to those achieved using a tyrosinase-reactive TCR. The latter approach, tested in an immunodeficient melanoma model was the first to demonstrate activity for adoptively transferred, antigen-specific Tregs (27). Both the tyrosinase and HLA-A2 encoding-genes are associated with vitiligo disease development, rendering relevance to tyrosinase-reactive T cells, as well as to the associated HLA-A*0201 restriction (28). The same combination of TCR and MHC transgenes was used to generate the h3TA2 mouse model of vitiligo, used here to host our CAR transgenic Tregs (29). These mice develop rapid, continuous depigmentation with most differentiated pigment cell lost from the skin by 5 weeks of age (30). This aggressive model of disease sets a high bar for therapeutics to be effective and can provide a first estimate of efficacy for new therapeutics (30). The ganglioside D3 (GD3) antigen, meanwhile, holds relevance as GD3 expression is found in stressed melanocytes, and likely more prominently so in perilesional skin of vitiligo patients (31). This GD3 antigen is currently targeted for immunotherapy of melanoma as for its expression on membrane and accessibility to antibodies (32, 33). Besides, GD3 contributes to melanogenesis, cell growth, and cell dendricity (34, 35). To test the therapeutic potential of antigen-specific Tregs for the treatment of vitiligo, we isolated naïve T cells and polarized them towards Tregs before introducing a GD3-responsive CAR-encoding construct. Resulting cells were tested for cytokine secretion to relevant targets *in vitro*, and resulting cytotoxicity was measured. Finally, in a mouse model of progressive vitiligo, we have introduced the resulting CAR-transgenic Tregs to test their treatment potential *in vivo*. This includes evaluating depigmentation, and the persistence of Tregs and melanocytes in the skin. Adoptive transfer of antigen-specific Tregs may offer an exciting opportunity to halt depigmentation and to complement up-and-coming therapeutics such as modified inducible HSP70 to tolerize dendritic cells (DCs) and Janus kinase (JAK) inhibitors to mitigate T cell activation (36, 37).

## Materials and Methods

### Study Design

The purpose of this study was to investigate the efficacy of GD3 CAR Tregs to halt progressing depigmentation in a mouse model of human vitiligo. We polarized and amplified Tregs *in vitro* before generating antigen-specific Tregs using a CAR construct to generate GD3 CAR Tregs with high transduction efficiencies. To define *in vitro* effects, we co-cultured human HLA-A2^+^ melanocytes along with tyrosinase reactive effector T cells in the presence of untransduced Tregs and GD3 CAR Tregs. For *in vivo* efficacy, we adoptively transferred untransduced Tregs, GD3 CAR Tregs, and HBSS vehicle into a humanized mouse model prone to develop vitiligo, subjecting the animals to depigmentation analysis, while evaluating the local and distant effects of CAR Tregs by immunohistology and multiplex cytokine analysis. Human skin from vitiligo patients were stained for GD3 expression on lesional, perilesional, and non-lesional skin.

Sample sizes for *in vivo* experiments were determined based on statistical power calculations from previous studies and past experience with the h3TA2 mouse model of vitiligo. For *in vivo* studies, mice were randomly assigned to groups with regard to sex, and investigators were blinded to the experimental conditions and to the further analysis. All the experimental samples and animals were included in the analysis, with no exclusion of outliers. Sample sizes, replicates, and statistical methods are indicated in the results and in the figure legends.

### Tissue Procurement, Cell Culture, and Reagents

Human perilesional skin tissue was obtained with informed consent from vitiligo patients attending the Dermatology clinic at Loyola University Medical Center in Maywood, IL (**Supplementary Table 1**). Studies were approved by the Institutional Review Board in adherence to principles described in the Declaration of Helsinki. Naïve mouse CD4^+^ T cells and CD4^+^ FoxP3^+^ Tregs were cultured in RPMI media supplemented with 10% FBS, 1X non-essential amino acids (Corning), 50 U/ml Penicillin-Streptomycin (Thermo Fisher Scientific), 1 mM Sodium Pyruvate (Gibco Life Technologies), 10 mM HEPES (Gibco Life Technologies), and 50 µM β-Mercaptoethanol (Sigma Aldrich). Human melanocytes were cultured in Human Melanocyte Growth Supplement-2 (Thermo Fisher Scientific) added to Medium 254 (Thermo Fisher Scientific) with 10 mM L-Glutamine (Thermo Fisher Scientific) and 1X antibiotic-antimycotic (Thermo Fisher Scientific). Rabbit anti-GD3 CAR sera and stable GD3 CAR virus producing cells were generated as described (38). Stable GD3 CAR virus producing cells (VPCs) were maintained in the above described T cell medium for virus production.

### Isolation of Naïve CD4+ T Cells and Polarization to CD4+FoxP3+ Tregs *In Vitro*

Naïve mouse CD4^+^ T cells were isolated from spleens of 8–10 week-old B6.Cg-*Foxp3^tm2Tch^*/J (“FoxP3 eGFP”) reporter mice (Jackson Laboratories) using EasySep Mouse naive CD4+ T cell isolation kit (StemCell Technologies) following the manufacturer’s protocol. These mice co-express enhanced green fluorescent protein (eGFP), which is restricted to the T cell lineage, primarily to the CD4^+^ T cell population. Naïve CD4^+^ T cells were polarized to CD4^+^ FoxP3^+^ using 30 ng/ml human transforming growth factor beta (TGF-β) (eBioscience) in the presence of Dynabeads™ Mouse T-Activator CD3/CD28 (Thermo Fisher Scientific) with a 1:1 bead to cell ratio and 300 international units/ml (IU/ml) of recombinant human interleukin 2 (rhIL-2) (NIH, Bethesda, MD) for 5 days. Human TGF-β was used to polarize murine Tregs as mouse and human TGF-β share 99% sequence homology with high cross-species activity (39, 40). Human IL-2 was used as human IL-2 efficiently stimulates mouse IL-2 receptor, whereas mouse IL-2 does not elicit efficient binding to human IL-2 receptors (41, 42).

### Generation of GD3 CAR Transduced Mouse Tregs

Twenty-four well non-tissue culture plates were coated with 10 μg/ml retronectin (Takara Bio USA Inc.). The MFG retroviral vector-based second generation CAR construct (sFv-CD28/TCRζ) reactive to GD3 was generated as described (43). Conditioned medium supernatant from Phoenix E retroviral producer cells (43) consisting of GD3 CAR-encoding virus (80% confluent) (43, 44) was transferred to retronectin coated plates and centrifuged at 2,000xg. Supernatant was carefully removed and activated CD4^+^ FoxP3^+^ Tregs were transferred to the retronectin coated plates with additional viral supernatant, 5 μg/ml protamine sulfate (Sigma Aldrich) and 300 IU/mL rhIL2. Plates were centrifuged at 1,000xg, before an incubation with complete T cell culture medium and mouse T-Activator CD3/CD28 beads and rhIL-2 as above. The transduction was then repeated to increase the transduction efficiency. Transduced Tregs were reactivated with CD3/CD28 beads, 30 ng/ml human TGF-β and rhIL-2 for 2 days before flow analysis.

### Flow Cytometry

Prior to surface staining, cells were incubated with mouse Fc Block (BioLegend) and LIVE/DEAD Fixable Near Infrared Dead Cell dye (Thermo Fisher Scientific) according to the manufacturer’s instructions. Surface staining of directly labeled antibodies included BUV395-labeled anti-mouse CD3 clone 145-2C11 (BD Biosciences), and BV421-labeled anti-mouse CD4 clone GK1.5 (BioLegend). The eGFP marker expressed under the FoxP3 promoter in Treg reporter mice, as well as BB700- labeled rat anti-mouse CD25 clone PC61 (BD Biosciences) were used to identify Tregs. Unlabeled anti-GD3 CAR rabbit sera detected by anti-rabbit allophycocyanin (APC) (Invitrogen) antibodies were used to evaluate CAR expression by transgenic Tregs. Stained cells were analyzed using a BD FACSymphony flow cytometer and FlowJo v10.3.0 software (FlowJo LLC, OR, USA).

### *In Vitro* Co-Culture Experiments

HLA-A2^+^ melanocytes were identified by immunofluorescent staining using FITC-labeled BB7.2 to human HLA-A2 prior to *in vitro* co-culture experiments. Human HLA-A2^+^ neonatal foreskin melanocytes (Mf0887, P6) and HLA-A2- abdominoplastic skin melanocytes (Ms18001, P6) were plated with tyrosinase reactive h3T effector T cells (Teffs) (29) and either untransduced or GD3 CAR-transduced suppressor Tregs at 10:1:1 effector to target to suppressor ratio for 36 h. Teff : Tregs ratio was used to mimic the natural occurrence of the T cell subsets as Tregs comprise 5–10% of the total T cell population Co-cultures were seeded in triplicates and incubated using IncuCyte^®^ Caspase-3/7 Red Apoptosis Assay Reagent (Sartorius). Images were taken every 3 h, in triplicate, using the IncuCyte live-cell analysis system (Sartorius). Supernatants were saved for mouse IFN-γ (R&D systems, Minnesota, MN) and IL-10 ELISA assay (Mabtech AB, Stockholm, Sweden) performed according to the manufacturer’s protocols. Cytotoxicity was examined by quantifying live cells relative to control wells using Adobe Photoshop (Adobe Systems, San Jose, CA).

### Adoptive Treg Transfer

Transgenic h3TA2 recipient mice with T cells expressing a TCR reactive to the human tyrosinase 368-376 (YMDTMSQV) epitope (21) were maintained under protocols approved by Northwestern University’s Institutional Animal Care and Use Committee (IACUC) following guidelines for the care and use of laboratory animals as outlined by the US National Research Council. Mice were retro-orbitally administered 2x10^5^ untransduced Tregs/per animal (n = 11; 6♂, 5♀) or 2x10^5^ GD3 CAR Tregs/per animal (n = 11; 6♂, 5♀), or treated with vehicle (HBSS) alone (n = 12; 6♂, 6♀) four times, every two weeks, starting at 5 weeks of age. The number of adoptively transferred Tregs was identified to enable a comparison to our earlier studies (21), where the 2x10^5^ polyclonal Tregs controlled depigmentation in in the human Tyrosinase TCR Transgenic- HLA-A2 (h3TA2) mouse model between 3–9 weeks old mice. All groups received low dose of recombinant human IL-2 (3,000 IU) 3 times a week throughout the entire experiment to promote *in vivo* stimulation of adoptively transferred Tregs (45, 46). Animals were maintained for 15 weeks and humanely euthanized. Experiments were initiated at 6 different time points, including mice from different litters. Naïve T cells were polarized and transduced to generate CAR Treg for up to 4 mice/group at a time. Depigmentation was monitored for each experiment over time, with results pooled to further substantiate the results. Skin biopsies, spleen, brain, ileum, lymph nodes were maintained in optimal cutting temperature (OCT), and serum was stored for cytokine analysis.

### Depigmentation Analysis

From 5 weeks to 15 weeks of age, mice were scanned weekly on a flatbed scanner (Hewlett-Packard, Palo Alto, CA) under isoflurane anesthesia. Using Adobe Photoshop software (Adobe Systems) ventral and dorsal luminosity was measured to calculate depigmentation, as previously described (47). Depigmentation was graphed over time, and statistical significance was determined by the time-adjusted area under the curve (AUC). Representing change in depigmentation from treatment initiation was calculated using the trapezoidal rule. No imputation was done for missing data, and the AUC for each mouse was divided by the total number of weeks of available data minus 1. The Wilcoxon Rank Sum (WRS) test was used to compare the time-adjusted AUC among groups.

### Immunohistology

Mouse and human skin samples were frozen using OCT Compound (Sakura Finetek) on dry ice. Eight µm cryosections were cut (Leica). For FoxP3/CD3 staining, sections were paraformaldehyde-fixed and permeabilized using True-Nuclear Transcription factor buffer set (BioLegend). Sections were treated with SuperBlock (ScyTek Laboratories, Logan, UT). PE-labeled antibody 145-2C11 to mouse CD3ϵ (Biolegend) and Alexa Fluor 488-labeled antibody MF-14 to mouse FoxP3 (BioLegend) were used for double staining procedures, followed by 4′,6-diamidino-2-phenylindole (DAPI) (BD Biosciences) nuclear staining. For other tissue stainings, mouse and human skin sections were fixed in cold acetone. Mouse skin sections were blocked with SuperBlock and then incubated with either antibody H-90 to TRP-1 (Santa Cruz Biotechnology, Dallas, TX) followed by Alexa Fluor 555 labelled donkey anti-rabbit antibody (abcam), or PE-labeled MB3.6 to GD3 (Santa Cruz Biotechnology), or PE-labeled antibody YGITR 765 to Glucocorticoid-Induced TNF Receptor (GITR) (Biolegend), or AF488-labeled antibody B56 to Ki67 (BD Biosciences), all followed by DAPI nuclear staining. Human skin sections were blocked with 10% normal human serum (Gemini Bio Products, West Sacramento, CA) and then incubated with Ta99 to TRP-1 (BioLegend) or R24 to GD3 (Abcam, Cambridge, UK). Both were detected by an HRP-conjugated goat anti-mouse IgG antibody (Agilent Dako, Santa Clara, CA). These stainings were developed using AEC substrate (Abcam) and nuclei were subsequently detected by incubation in Mayer’s hematoxylin (Sigma-Aldrich) and blued in Scott’s tap water (Sigma-Aldrich). Slides were imaged on a Revolve microscope (Echo Laboratories). Cells were quantified using Adobe Photoshop software.

### Cytokine Analysis

Included in cytokine analysis were supernatants from *in vitro* suppression assays (IncuCyte experiments), collected 36 h post-co-culture, and serum samples from HBSS vehicle (n = 11), untransduced (n = 10), and GD3 CAR Tregs (n = 9) treated mouse groups. Detection of murine Interferon gamma (IFN-γ), tumor necrosis factor (TNF-α), IL-4, and IL-10 was performed by using a custom made V-Plex panel for these mouse cytokines (Meso Scale Diagnostics, LLC) according to manufacturer’s instructions. Data were acquired on MESO Quickplex SQ120 (Meso Scale Diagnostics, LLC) and analyzed using Prism version 8.3.0 (GraphPad Software).

### Statistical Analysis

Statistical analysis was performed using GraphPad Prism 8.0 software (GraphPad) and R-software. Data are presented as bars and dot plots with mean values ± standard deviation. The data were evaluated by one-way analysis of variance (ANOVA) analysis of variance accounting for different variances across the treatment groups, with post-hoc Tukey-Kramer comparisons. To determine statistical significance for immunosuppression *in vitro*, two-way ANOVAs were used with aligned rank transformation followed by multiple pairwise comparison testing using Tukey approach. For depigmentation, the time-adjusted AUC, representing change in depigmentation from treatment initiation, was calculated using the trapezoidal rule. No imputation was done for missing data, and the AUC for each mouse was divided by the total number of weeks of available data minus 1. The WRS test was used to compare the time-adjusted AUC among groups. Statistical significance is represented as *p < 0.05, ** p < 0.01, *** p < 0.001, or **** p < 0.0001.

## Results

### GD3 Is Expressed by Perilesional Epithelial Cells Including Melanocytes

**Figure 1:**
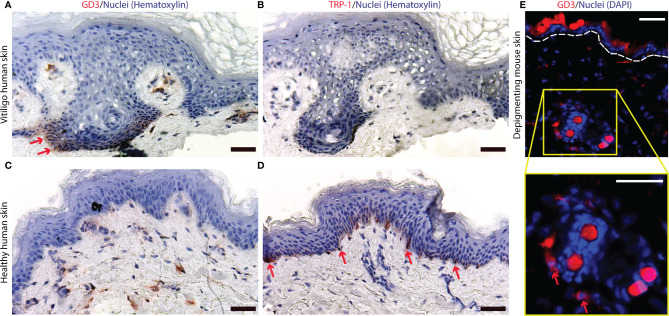
Ganglioside D3 is expressed in depigmenting skin of human and mouse origin. **(A)** Expression of GD3 and **(B)** a lack of melanocytes, as represented by an absence of TRP-1+ staining, were found in human depigmenting epidermis from perilesional patient skin. Whereas **(C)** limited GD3 expression and **(D)** abundant TRP-1 expression are shown in healthy control skin. **(E)** In depigmenting mouse skin, GD3 expressing cells were observed around hair follicles and in proximity to the epidermis. Representative staining of GD3 expressing cells is shown in red with nuclei in blue, around autofluorescent mouse hairs (Scale bar = 50μm).

Overexpression of O-acetylated GD3 has been reported for actively depigmenting vitiligo skin (31, 38). This prompted us to evaluate GD3 expression itself in skin biopsies from perilesional biopsies taken from actively depigmenting skin. Marked expression of GD3 was observed in human vitiligo perilesional epidermis (**Figure 1A**), while melanocytes are absent from the border biopsy section shown (**Figure 1B**). Vacuolization in **Figure 1A** and **1B** is frequently observed in vitiligo skin, and has been proposed as an indication of vitiligo by others (48, 49). Epidermal GD3 expression was not observed in healthy control skin (**Figure 1C**) whereas melanocytes are readily detectable in this tissue (**Figure 1D**). Similarly, GD3 expression was found in depigmenting h3TA2 mouse skin (**Figure 1E**).

### High Viral Transduction of Tregs Was Achieved With GD3-Encoded CAR Construct

**Figure 2:**
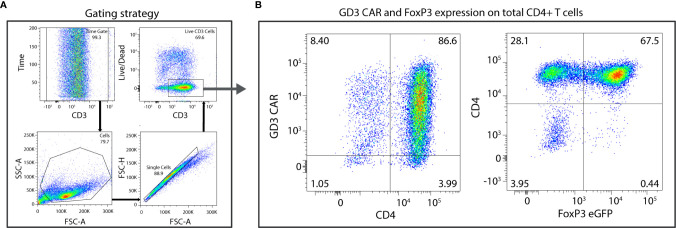
High transduction efficiencies are observed for Tregs expressing the GD3 CAR. CD4^+^FoxP3^+^ cells, polarized from naïve CD4^+^ T cells, were transduced using a GD3 CAR-encoding construct. **(A)** The gating strategy consists of a time gate followed by sequentially gating on lymphocytes, single cells, and live cells. **(B)** Eighty-six percent of total CD4^+^ T cells were successfully transduced to express the GD3 CAR construct and 67% of that population express FoxP3^+^.

To generate therapeutic Tregs that will engage in suppressive activity where needed, we generated FoxP3^+^CD4^+^ T cells and transduced them to express a GD3-reactive CAR. In a representative example, approximately 1.5x10^6^ naïve CD4^+^ T cells were isolated from 3x10^8^ splenocytes, maintained in presence of TGF-β, and successfully polarized and amplified to approximately 1.6x10^7^ Tregs per donor mouse. TGF-β-polarized naïve CD4^+^ T cells were retrovirally transduced and GD3 CAR expression was evaluated by flow cytometry. The gating strategy is shown in **Figure 2A**, where 86.6% of total CD4^+^ T cells were successfully transduced with the GD3 CAR construct (**Figure 2B**). After further expansion, 64 ± 3.5% transduced cells were FoxP3^+^ Tregs. From an initial pre-expansion and transduction pool of 4x10^6^ FoxP3^+^ Tregs, 2.1x10^7^ GD3 CAR-expressing, FoxP3^+^ Tregs were generated. The majority of resulting CAR transduced Tregs are expected to function as immunosuppressive T cells, and exert a local, immunosuppressive function. We next measured GD3 CAR Treg function *in vitro*.

### Antigen Specificity Increases Immunosuppressive Cytokine Production

**Figure 3:**
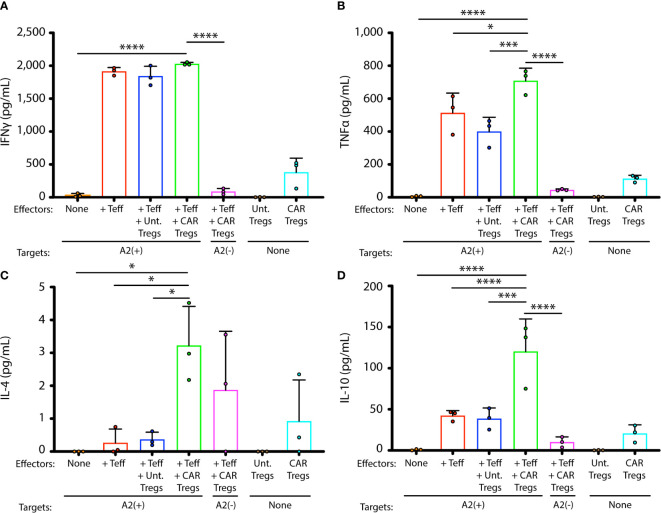
GD3 CAR Tregs generate immunosuppressive cytokines in presence of activated T cells. Cytokines were measured in supernatants from cocultures of melanocyte targets and HLA-A2-restricted Teffs, in presence and absence of untransduced or CAR-transduced Tregs. Cytokine concentrations for each coculture, measured in triplicate cocultures, are shown for **(A)** IFN-γ, **(B)** TNF-α, **(C)** IL-4, and **(D)** IL-10. Statistical analysis was performed by a one-way ANOVA test followed by Tukey’s post-hoc test for multiple comparisons. *p < 0.05; **p < 0.01; ***p < 0.001; ****p < 0.0001.

Production of representative cytokines IFN-γ, TNF-α, IL-4, and IL-10, relevant to immune activation or immunosuppression, was measured in co-cultures of GD3 CAR Tregs or untransduced Tregs with tyrosinase-reactive Teffs and their HLA-matched targets (1:10:1), measuring concentrations 42 h after cells were combined in culture in presence of IL-2 (**Figure 3**). Human melanocytes can be recognized by these Teffs (29). No significant differences in IFN-γ production were found in combinations that do or do not contain Tregs, suggesting that the latter had little influence on the production of this cytokine at this Treg to Teff ratio (**Figure 3A**). Significantly more TNF-α (**Figure 3B**, p = 0.0005), IL-4 (**Figure 3C**, p = 0.03), and IL-10 (**Figure 3D**, p = 0.0005) was produced in combinations with CAR Tregs, though overall IL-4 production remained consistently low. Importantly, increased IL-10 regulatory cytokine production was observed only in presence of cytotoxic T cells and HLA-matched human melanocytes. Taken together, the cytokine environment suggests a greater immunosuppressive ability in presence of antigen specific Tregs, stimulated by activated effector T cells. To measure whether this cytokine environment might translate to greater protection of melanocyte target cells from cell death *in vitro*, we next measured sustained target cell viability in these cocultures of melanocytes, Teff and Tregs.

### Antigen Specificity Increases the Immunosuppressive Activity of Tregs *In Vitro*

**Figure 4:**
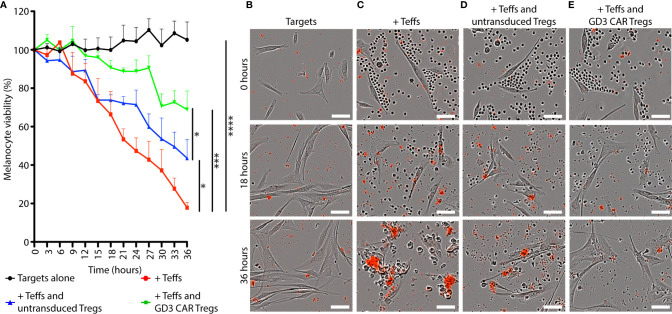
GD3 CAR Tregs provide melanocytes with superior protection from T cell-mediated cytotoxicity *in vitro*. The immunosuppressive ability of GD3 CAR Tregs and untransduced Tregs was compared *in vitro.* ***(*A*)*** Viability of HLA-A2^+^ human melanocytes (targets) in the presence or absence of murine Teffs and Tregs (1:10:1) is represented over time. Representative images of **(B)** HLA-A2^+^ human melanocytes **(C)** combined with murine Teffs, and additionally with **(D)** murine untransduced Tregs, or **(E)** GD3 CAR Tregs. Dead cells are marked by a red precipitate formed by caspase activity. Statistical significance was determined by two-way ANOVA with aligned rank transformation followed by Tukey’s pairwise multiple comparisons test. *p < 0.05; **p < 0.01; ***p < 0.001; ****p < 0.0001 (Scale bar = 50μm).

Tregs suppress conventional T cells *via* cytokines, by cell-to-cell contact or through bystander effects (50). To measure the resulting suppressive activity, we evaluated sustained melanocyte viability in co-cultures of targets, Teffs, and Tregs *in vitro* for 36 h. **Figure 4A** shows the viability of targeted HLA-A2^+^ human melanocytes in different combinations of targets, Teffs and Tregs 1:10:1. The number of viable targets increased slightly over time in absence of Teff cells. In comparison, 82.2% cytotoxicity (p < 0.0001) was observed in presence of effector T cells after 36 h. Untransduced Tregs offered 35.8% (p = 0.02) protection from cytotoxicity over time. A two-way ANOVA was performed with aligned rank transformation using R-software, and pairwise post-hoc multiple comparison testing according to Tukey to determine that in presence of CAR Tregs, cytotoxicity towards melanocytes was 62.0% prevented (p = 0.0004). Images representing each combination of cells including targets alone (**Figure 4B**), targets and Teff (**Figure 4C**), and the latter combination in presence of untransduced Tregs (**Figure 4D**) or CAR Tregs (**Figure 4E**) at different time points likewise reveal most inhibition of cytotoxicity in a combination that includes GD3 CAR Tregs. Thus, both untransduced Tregs and GD3 CAR Tregs offered significant protection of melanocyte viability. Importantly, the protection offered by GD3 CAR Tregs was significantly greater compared to untransduced Tregs *(*p = 0.04*)*, demonstrating the added benefit of antigen specificity to enhance immunosuppression. Thus, we next explored the therapeutic effects of CAR Tregs *in vivo*.

### Antigen-Specific Tregs Enhance Immunosuppression in h3TA2 Mice

**Figure 5:**
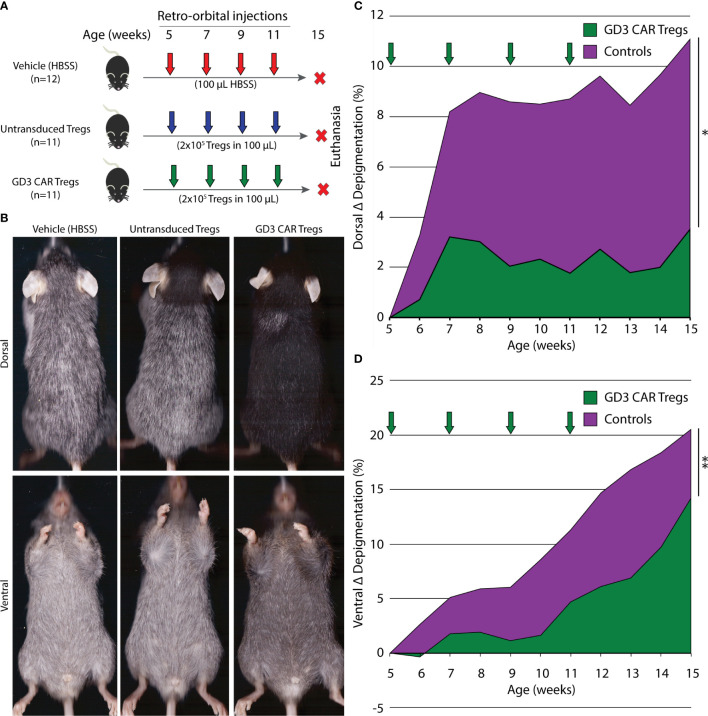
GD3 CAR Tregs provide significant protection from depigmentation in vitiligo-prone mice. **(A)** Experimental outline initiated at 6 different time points showing vitiligo prone, h3TA2, mice treated with vehicle alone (n = 12), or by adoptive transfer of untransduced Tregs (n = 11) or GD3 CAR Tregs (n = 11). Adoptive transfer started at 5 weeks of age and continued biweekly until 11 weeks of age. Depigmentation was measured weekly from 5–15 weeks of age. **(B)** Representative dorsal and ventral scans of mice from the HBSS vehicle, untransduced Treg, and GD3 CAR Treg treated groups at 15 weeks of age. **(C)** Depigmentation quantified on dorsal and **(D)** ventral sides throughout the experiment. The Wilcoxon rank sum (WRS) test was used to compare the time-adjusted AUC among groups. Arrows: treatment times. *p < 0.05; **p *<* 0.01.

To evaluate the suppressive activity of CAR Treg in a model of progressive depigmentation, we measured depigmentation in spontaneously depigmenting h3TA2 mice starting from 5 weeks of age. Depigmentation starts shortly after birth and the animals display half-maximum depigmentation within 23 weeks (51). Mice received adoptively transferred untransduced Tregs, transduced GD3 CAR Tregs or vehicle once every two weeks for 11 weeks as outlined in **Figure 5A**. Representative dorsal and ventral images of animals transfused with untransduced Tregs, GD3 CAR Tregs, or vehicle are shown in **Figure 5B**. The Wilcoxon rank sum (WRS) test was used to compare the time-adjusted area under the curve (AUC) among groups. Outcomes for both vehicle and untransduced Treg control groups did not differ (dorsal p = 0.97, ventral p = 0.88). Therefore, the vehicle and untransduced Treg groups were merged, and compared to the GD3 CAR Treg-treated group. In a one-sided t-approximation for the WRS test, the AUC for dorsal depigmentation dropped by 73.0% (p = 0.028) for CAR Treg treated mice (n = 11) for the 15-week observation period. Ventral depigmentation occurs more rapidly and was evaluated separately. Here, depigmentation was significantly delayed among the CAR Treg treated group (n = 11) over the follow-up period (**Figure 5C**) resulting in a 60.5% reduction in the AUC (p = 0.006) among CAR Treg treated mice (**Figure 5D**). Individual dorsal and ventral depigmentation values for each mouse are shown in the supplementary data file (**Supplementary Figure 1**). The enhanced disease control by CAR Tregs might be due to local activation of suppressive activity by GD3 expression and the presence of activated Teff on site. To assess this, changes in serum cytokine content for IFN-γ, TNF-α, IL-4, and IL-10 were measured in serum samples from mice treated with vehicle alone (n = 11), untransduced Tregs (n = 10), or GD3 CAR Tregs (n = 9). Resulting cytokine levels were remarkably consistent among the groups at end point (**Supplementary Figure 2**). The results support the concept that Tregs, including CAR Tregs, may be preferentially activated on site in areas of immune activity. Adverse events were not observed throughout the experiment, and no abnormalities were found during internal organ examination at euthanasia for mice from any groups. We next probed whether reduced depigmentation was accompanied by a sustained presence of melanocytes and changes in T cell populations.

### Melanocytes Are Protected in the Presence of GD3 Reactive CAR Tregs

**Figure 6:**
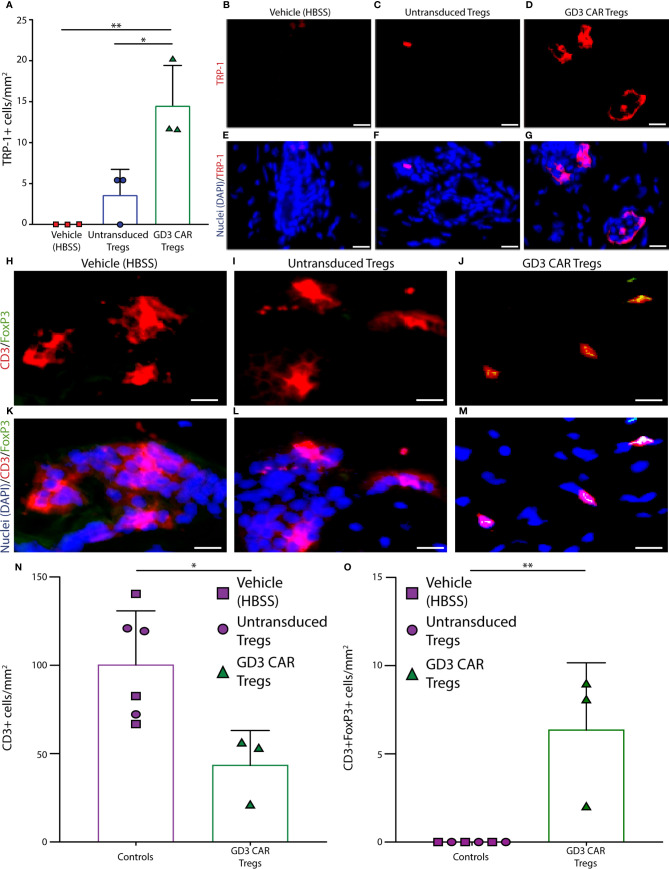
Melanocytes are protected from h3T cytotoxic T cells in the presence of GD3 reactive CAR Tregs. Mouse skin was evaluated for melanocyte presence using antibodies to TRP-1. **(A)** Quantification of melanocytes, as well as accompanying representative images of TRP-1 staining from **(B)** HBSS vehicle, **(C)** untransduced Tregs, and **(D)** GD3 CAR Tregs treated mice (n = 3 per group), with **(E–G)** the respective overlay including DAPI nuclear staining in blue is shown. Mouse skin tissues were also evaluated for T cell infiltration using antibodies to CD3ϵ and FoxP3, and examples of staining in skin from **(H)** vehicle control, **(I)** untransduced Tregs, and **(J)** GD3 CAR Tregs administered mice are shown. Representative samples were used to quantify CD3ϵ^+^ T cells (red), FoxP3^+^ cells (green), and double positive Tregs; **(K–M)** respective overlays with DAPI (blue) are also shown. Quantification of skin staining ± SD (*n* = 3 per group) for **(N)** T cells and **(O)** Tregs are shown, respectively. Statistical analysis was performed by non-parametric t tests. *p *<* 0.05, **p < 0.001 (Scale bar = 20 μm).

Mouse dorsal skin biopsies were evaluated for melanocyte abundance using antibodies to TRP-1, as shown in **Figure 6**. Melanocytes were quantified as shown in **Figure 6A**, where skin samples from vehicle treated mice (n = 3 per group) showed complete loss of melanocytes. Skin from untransduced Treg treated mice (n = 3 per group) displayed only a few remaining melanocytes, and a one-way ANOVA was performed followed by Tukey’s post-hoc test to demonstrate that whereas skin from CAR Treg treated mice contained a significantly greater number of melanocytes compared to mice treated with untransduced Tregs (p = 0.025), and to vehicle treated controls (p = 0.006). Representative images of TRP-1 staining for vehicle-treated, untransduced Treg-treated, and CAR Treg-treated mice are shown in **Figures 6B-D**, and overlaid with DAPI nuclear staining in **Figures 6E–G**, respectively. Similar results were found when examining GD3 expression. Quantification of GD3 expressing cells revealed that mice transfused with CAR-Tregs maintained significantly more GD3 expressing cells than the vehicle HBSS-treated mice (p = 0.003) or mice transfused with untransduced Treg (p = 0.003) (**Supplementary Figure 3**). This observation supports the concept that GD3 expressing cells did not experience the cytotoxicity observed in vehicle-treated or untransduced Treg treated mice. This confirmatory melanocyte quantification mainly corresponds with *in vivo* data shown in **Figure 6**, demonstrating the improved suppressive ability of CAR Tregs. To explain the differences in pigmentation and melanocyte maintenance, we next compared these data to Treg infiltration in each treatment group.

### CAR Tregs Gravitate Towards GD3 Expressing Cells in the Skin

**Figure 7:**
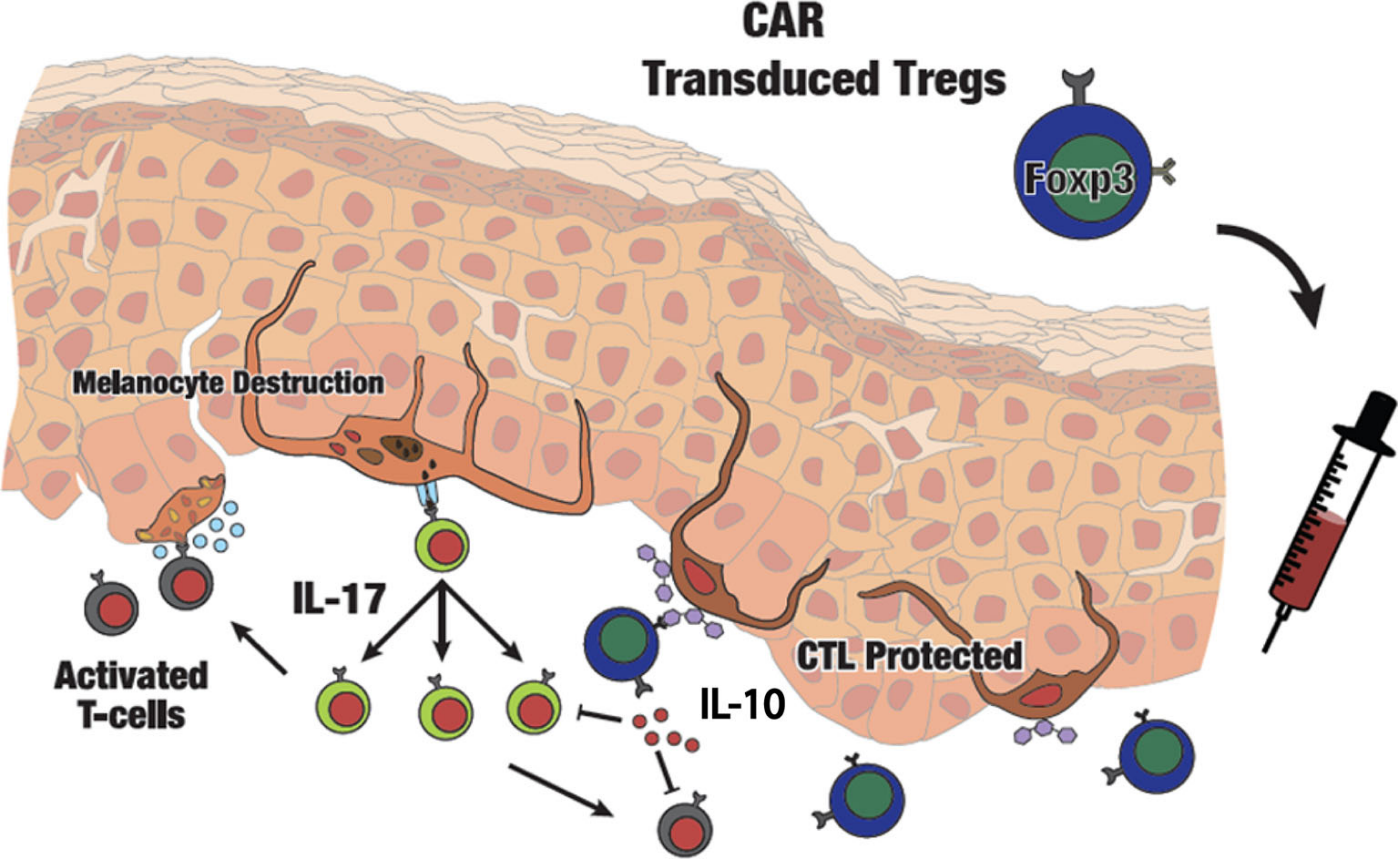
Schematic presentation of adoptive transfer of CAR Tregs in vitiligo. Autoimmune melanocyte destruction is mediated by cytotoxic T cells, which are activated *via* self-antigens secreted by stressed melanocytes. Elevated IL-17 promotes an inflammatory environment in the skin. Infused GD3-specific CAR Tregs will infiltrate the skin and respond to GD3 expressed at the site of autoimmune activity, suppressing cytotoxic T cells on site and providing local immune tolerance in vitiligo perilesional skin.

To understand whether Treg activity is correlated to the abundance of immunosuppressive T cells on site, mouse skin tissues were evaluated for T cell infiltration using antibodies to CD3ϵ and FoxP3. Examples of skin from the vehicle control group, and samples from the mice treated with untransduced or CAR Treg-treated mice are also shown in **Figure 6**. Tregs were identified as CD3ϵ^+^ FoxP3^+^ cells for the same groups in **Figures 6H–J**, respectively, overlaid with DAPI nuclear staining in **Figures 6K–M**. CD3ϵ^+^ cell and CD3ϵ^+^/FoxP3^+^ Treg abundance was quantified as the mean ± SD (at n = 3 per group) for each treatment group. In a one-way ANOVA followed by Tukey’s post-hoc test, the average number of infiltrating CD3ϵ^+^ T cells at end point was 2.3-fold greater (p = 0.02) in the control groups as compared to the CAR Treg treated group (**Figure 6N**). No (remaining) CD3^+^FoxP3^+^ Tregs were detected in either control group, whereas some CD3ϵ^+^ FoxP3^+^ Tregs were still detectable in skin tissue from CAR Treg treated mice 10 weeks after adoptive transfer (**Figure 6O**). Evaluating Treg numbers by GITR-expression, an increase in Treg numbers at end point was again observed in skin from CAR Treg treated mice compared to those treated with untransduced Tregs (p = 0.0059) or vehicle alone (p = 0.0089), yet there was no difference in abundance of proliferating GITR^+^Ki67^+^ cells among groups. This suggest that differences in Treg abundance may instead be defined by increased influx or decreased efflux of Tregs from the skin in CAR Treg treated mice (**Supplementary Figure 4**). Nevertheless, the increased abundance of Tregs in CAR Treg treated mice at end point may explain the improved suppressive activity by CAR Tregs and suggests that maintenance of a Treg presence on site is supported by local antigen recognition (**Figure 7**). In summary, the data show that antigen specificity prolonged the suppressive activity of adoptively transferred Tregs.

## Discussion

Here we describe the therapeutic potential of engineered GD3 CAR Tregs to provide antigen-specific immune tolerance for autoimmune vitiligo. In contrast to conventional immunosuppressive drugs, biologics, alkylating agents, and antimetabolites, Tregs can provide greater specificity with complex therapeutic benefits, and restore immune tolerance in various autoimmune diseases (20). Preclinical studies have provided promising results when using polyclonal CD4^+^CD25^+^FoxP3^+^ Tregs to enforce immune tolerance in various mouse models, including vitiligo (21). A robust human Treg isolation protocol was established to sort Tregs with >90% purity, and an *ex vivo* expansion protocol was developed to acquire upwards of 3x10^9^ Tregs from a single donor, similar to the protocol used here (52). Overall, this and other phase I clinical studies provided answers to the isolation, and expansion that surrounded Treg immunotherapy (53–56). Yet the efficacy of polyclonal Treg transfer is not self-evident to date.

In vitiligo, some studies reported no significant differences in circulating Treg numbers (18, 57), whereas others report a difference in Treg abundance between vitiligo patient and healthy blood (15, 58). Whereas circulating Treg numbers may vary, more consistency is found among reports of a local deficiency of Tregs in patient skin, which might be a cause for uncontrolled peripheral immune responses and progressive depigmentation in vitiligo (12, 18, 57).

Adoptive transfer of islet-specific Tregs outperformed polyclonal Tregs for blocking type 1 diabetes progression compared to polyclonal Tregs (20, 59, 60). Unfortunately, diabetes is generally detected in patients when pancreas destruction is near complete, and other conditions may be more amenable to Treg based treatment in a clinical setting. Preclinical studies likewise suggest a superior efficacy of antigen-specific Tregs in transplantation procedures (61–64).

In the h3TA2 mouse model of vitiligo, new Teff are continuously attacking melanocytes, thus reflecting continuously active disease. In these mice, Tregs might be effective for the duration of treatment whereas depigmentation will return when adoptive transfer is halted. Indeed, adoptive Treg treatment is expected to be most efficacious during active disease. In human patients however, progressive disease periods are interspersed with periods of inactivity, providing melanocyte stem cells with an opportunity to differentiate and repopulate the depigmented lesions. Repigmentation can occur in patients treated with JAK inhibitors to suppress Teff activity, though supportive treatment by UV light may be required (65). For intermittent treatment of vitiligo, it will be beneficial to store autologous GD3 CAR Tregs for later use (66).

By virtue of their antigen specificity, these Tregs might provide better safety profiles and decrease the risk of generalized immunosuppression. This is supported by increased IL-10 secretion found where Tregs function in presence of activated Teff, as shown here. When comparing Tregs expressing first and second generation CARs, cells expressing a second-generation CAR with a 28ζ costimulatory domain the greatest amount of IL-10 (67). The CAR Treg construct used to transduce the Tregs included in our paper similarly showed significant IL-10 production upon activation through the CAR, which might explain the improved suppressive activity of these GD3 CAR Tregs compared to untransduced Tregs *in vitro.* Indeed, TCR and CAR transduction may also produce more potent and stable Tregs for *in vivo*, clinical use in vitiligo, and this condition holds an important advantage for investigating the superiority of antigen-specific Tregs by offering several target antigens associated with the condition (68). TNF-α was significantly elevated both in culture and in serum of experimental mice. Nevertheless, genetic ablation of TNF-α was not correlated with the development of vitiligo, and no difference in depigmentation was found when compared to wild type h3TA2 mice (21). In vitiligo, TNF-α plays a role in the development cytotoxic T cells (CTLs) and enhances expression of IFN-γ, which are implicated in imitation of vitiligo development (21, 69, 70). Recent studies reports that TNF-α might promote anti-inflammatory conditions *via* activation and induction of Treg proliferation *in vivo* (71–73). Thus, TNF-α can potentially be both destructive and protective in vitiligo, by promoting CTLs and stimulating Tregs, respectively. While TNF-α depletion halted the disease progression and promoted repigmentation in vitiligo, 18 of 5,928 patients developed vitiligo *de novo* when TNF-α inhibitors were administered for other autoimmune disorders. This leaves anti-TNF-α treatment option for vitiligo until side effects are fully averted (74).

We thus asked whether antigen-specific T regs might provide additional benefit for the treatment of the autoimmune disease vitiligo. This condition holds a complex etiology (2), with melanocyte loss as a common denominator and immune mechanisms held universally responsible for the spread of disease. TCR- and CAR-based Tregs possess different mechanistic and functional properties. Low-antigen expression levels are sufficient for TCR-based Tregs to become activated, whereas CAR-based Tregs require high density of antigens (75, 76) suggesting that TCRs or CARs could be selected based on antigen expression by target tissue. We performed repeat Treg injections, because the mice in our model exhibit chronically active disease and we do not yet know how long transgenic Tregs remain active on site. The long-term fate of adoptively transferred Treg has yet to be established to better understand the need for repeated applications. Importantly, CARs are not subjected to HLA restriction and hold a higher affinity for their targets moieties (77). Thus, a CAR construct can find a universal application for patients with progressive disease.

We identified a potential target for antigen-specific Tregs in vitiligo. Our GD3 CAR Tregs protect melanocytes from T cell-mediated destruction in a mouse model of vitiligo, expressing a human TCR and matched human MHC, and capable of responding to human target cells. Importantly, the antigen of choice does not need to be expressed by target cells spared in the response themselves (78).

Though no side effects were apparent in our current studies during internal organ examination, some safety concerns remain, including the possible development of cytokine release syndrome (CRS) or neurotoxicity if transduced cells ultimately develop an effector profile (79). CRS is however more likely to develop when targeting liquid tumors than in solid tissues (80). To counter any potential side effects and promote safety, a construct that includes a caspase-based suicide gene can be incorporated in order to inactivate the GD3 CAR Tregs if necessary (81, 82).

A limitation to intravenous injection of antigen specific Tregs might be that these much-needed immunosuppressive cells display a paucity at the desired site (60). Should systemically applied Tregs not respond as required, local injection might be needed, or the CCR4 Treg homing receptor ligand CCL22, can be introduced where Tregs are needed to attract systemically applied Tregs (17). This leaves autoimmune diseases of the skin especially suited for adoptive treatment by antigen-specific Tregs when relevant antigens can be identified. In fact, GD3 might support keratinocyte proliferation while O-acetylated GD3 was overexpressed in psoriatic skin (83, 84). These findings suggest that GD3 CAR Tregs might temper lesions in the latter condition as well.

To date, only alloantigen-reactive Tregs are currently being tested to prevent rejection after organ transplantation in clinical trials (60). Here we prepared and expanded GD3 reactive CAR Tregs in amounts suited for adoptive cell transfer therapy in mice. The same can be pursued in patients, cryopreserving the therapeutic Tregs for future use (85–88). One of the challenges of adoptive transfer is the cost and scalability of the technique. This has prompted the concept of developing off-the-shelf “Universal CAR Tregs” readily adaptable for all patients. Versatility is provided by modules that bind both the universal CAR and the target cell (89). In summary, the data provided here support the use of antigen-specific CAR Tregs as an adoptive cell therapy for vitiligo, to control depigmentation and support immune tolerance in vitiligo.

Treg infusion has thus far been well tolerated in patients. The results from currently ongoing clinical trials can bring important insights regarding the optimal Treg dose, expected efficacy and any concern that may arise about possible side effects, and the treatment strategy can be further adjusted to support continued maintenance and memory formation (90), improved homing (91), and safety measures to eliminate adoptively transferred cells (92) as needed. The efficacy and specificity of Treg therapy for conditions other than vitiligo can be enhanced where antigens can be identified to serve as targets for engineered, antigen-specific Tregs. *In vivo* tracking will allow research groups to better understand the maintenance and memory-forming potential of antigen-specific Tregs. In conclusion, Treg-based therapy holds potential as a future therapy for vitiligo and for other autoimmune skin diseases.

## Data availability statement

The original contributions presented in the study are included in the article/**Supplementary Material**. Further inquiries can be directed to the corresponding authors.

## Ethics statement

The animal study was reviewed and approved by Northwestern University’s Institutional Animal Care and Use Committee. Written informed consent was obtained from the individual(s) for the publication of any potentially identifiable images or data included in this article.

## Author Contributions

Conceptualization: CLP and ZM. Methodology: ZM, CLP, CC, and DJ. Formal Analysis, ZM. Investigation, ZM, ERD, JME, and KP-M. Statistical analysis, AI and KC. Resources, RPJ, SM and AO. Writing—Original Draft, ZM. Writing—Review and Editing, ZM, CLP, ERD, YOO and JRL. Visualization, ZM, ERD, JME and SWH. Supervision, CLP. Project Administration, CLP. All authors contributed to the article and approved the submitted version. 

## Funding

This study was supported in part by NIH RO1s AR057643, CA191317, and by The LAM Foundation through an Established Investigator award to CLP. A foreign internship to ZM was supported by the Ministry of Education and Science of the Republic of Kazakhstan under the Ph.D. program at Al-Farabi Kazakh National University.

## Conflict of Interest

United States and PCT international patent (Utility&PCT, serial No.17/072,939&PCT/US2020/056104) filed as “Materials and methods for treating vitiligo” on 10/16/2020.

The authors declare that the research was conducted in the absence of any commercial or financial relationships that could be construed as a potential conflict of interest.
