# Supplementary material for: Antigen Specificity Enhances Disease Control by Tregs in Vitiligo
Source: Front Immunol. 2020 Dec 1;11:581433. doi: 10.3389/fimmu.2020.581433 (PMC7736409; doi:10.3389/fimmu.2020.581433)
Supplement: Supplementary file 1 [file DataSheet_1.pdf]

Supplementary Table 1. Characteristics of patients who donated vitiligo skin for this study.

Biopsy site	Gender	Duration of vitiligo	Disease progression at the site of biopsy	Treatment
Elbow	Adult male	2 years	Progressing	Laser therapy
Buttock	Adult female	30 years	Progressing	Laser therapy
Hip	Adult female	15 years	Progressing	Laser therapy

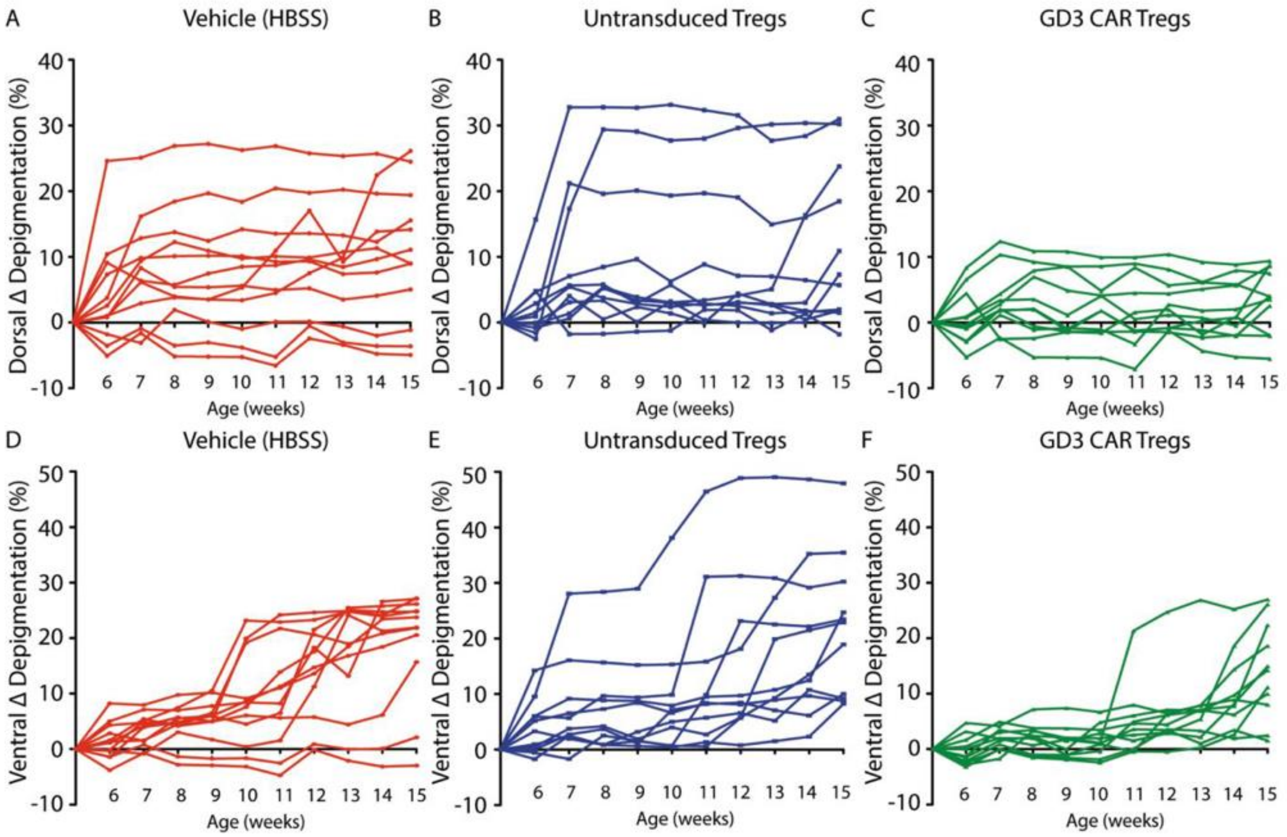

Supplementary Figure 1. Individual mouse depigmentation values over time support the treatment effects of CAR Tregs. Dorsal depigmentation, represented as the change from baseline over time, is shown for each individual mouse from the (A) vehicle treated (n=12), (B) untransduced Treg (n=11), and (C) GD3 CAR Treg (n=11) treated groups. Respective ventral depigmentation values for (D) vehicle, (E) untransduced Tregs and (F) CAR Treg are also presented.

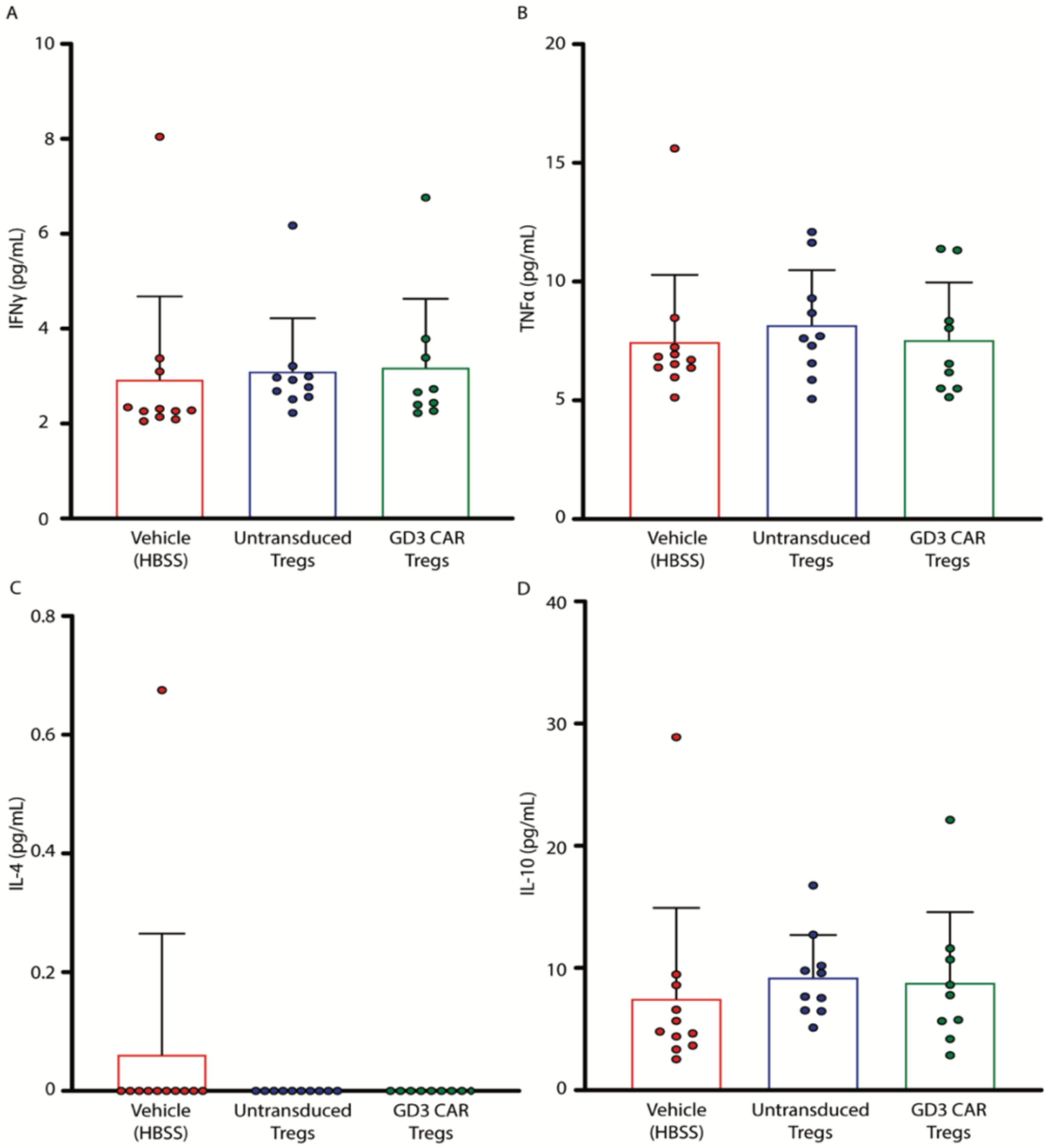

Supplementary Figure 2. No differences in relevant cytokine titers were observed in serum from experimental animals. Detection of (A) IFN-γ, (B) TNF-α, (C) IL-4 and (D) IL-10 in serum harvested at 15 weeks of age from vehicle treated (n=11), untransduced Treg treated (n=10), and GD3 CAR Treg treated (n=9), vitiligo-prone mice. Murine cytokine levels were unchanged between groups with $p < 0.05$ by one-way ANOVA followed by a Tukey post-test to correct for multiple comparisons.

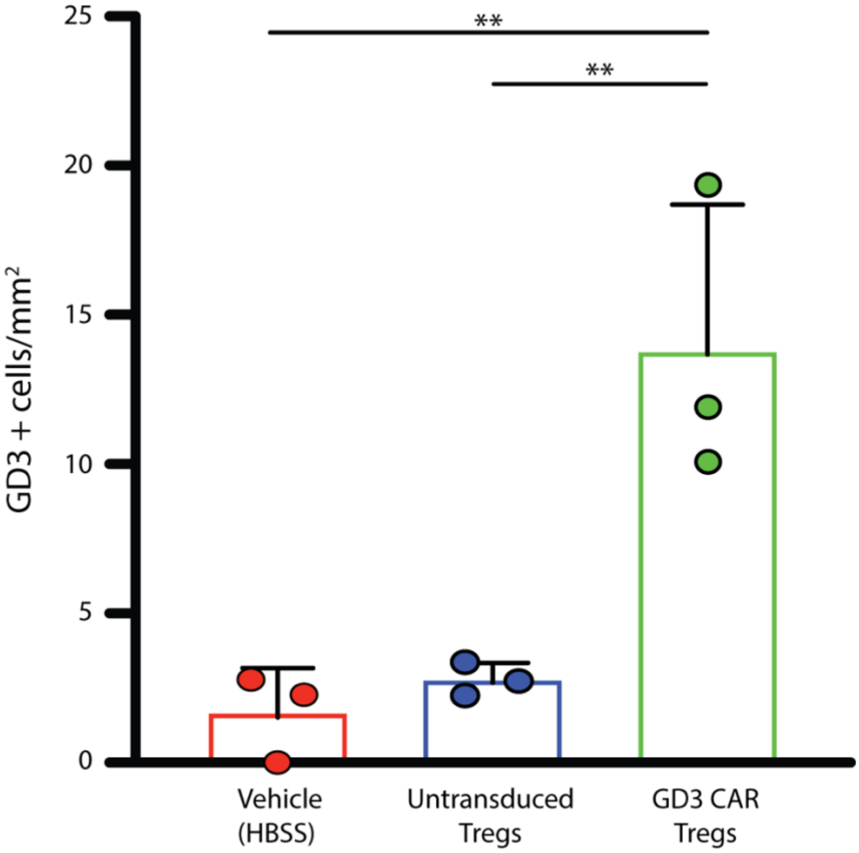

Supplementary Figure 3. Treg transfusion helps maintain GD3 expressing cells in h3T-A2 vitiligo mouse skin. Quantification of GD3 expressing cells from h3T-A2 mouse skin at end point (mean ± SD) is compared across recipients of vehicle treatment, adoptive transfer by untransduced Tregs, or by GD3 CAR Tregs (n=3 per group). Statistical significance was determined by one-way ANOVA followed by a Tukey post-test to correct for multiple comparisons *$p< 0.05$; **$p < 0.01$.

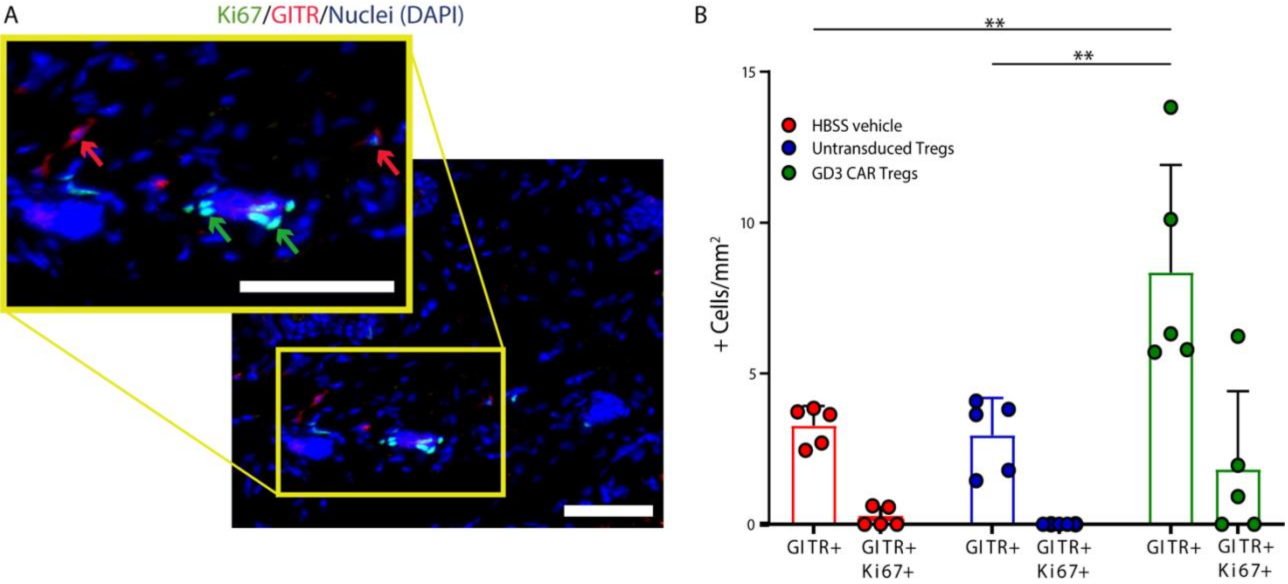

Supplementary Figure 4. Proliferation of GITR+ Tregs is not increased in the skin of CAR Treg recipient mice at 15 weeks. Cells expressing Ki67 (green) and GITR (red) in the skin of vitiligo-prone h3T-A2 mice were quantified at 15 weeks of age (mean ± SD) and compared among recipients of vehicle treatment, of untransduced Tregs, or of GD3 CAR Tregs (n=5 skin samples per group). Statistical significance was determined by one-way ANOVA followed by a Tukey post-test to correct for multiple comparisons. *p< 0.05; **p < 0.01 (Scale bar = 50μm).
